# Effect of induced hindlimb length difference on body-mounted inertial sensor measures used to evaluate hindlimb lameness in horses

**DOI:** 10.1371/journal.pone.0228872

**Published:** 2020-02-18

**Authors:** Jael B. Pitts, Joanne Kramer, Shannon K. Reed, Paul Schiltz, Lori Thombs, Kevin G. Keegan

**Affiliations:** 1 Veterinary Health Center, Department of Veterinary Medicine and Surgery, Equine Surgery, College of Veterinary Medicine, University of Missouri, Columbia, Missouri, United States of America; 2 Equestrian Studies, William Woods University, Fulton, Missouri, United States of America; 3 Social Science Statistics Center, Department of Statistics, University of Missouri, Columbia, Missouri, United States of America; West Virginia University, UNITED STATES

## Abstract

This study has investigated the immediate effect of induced hindlimb length difference on hindlimb lameness measured as differences in minimum (Pmin) and maximum (Pmax) pelvic heights in 16 horses trotting in a straight line and lungeing on both hard and soft surfaces with body-mounted inertial sensors. Hindlimb length differences were induced by applying an Easyboot Glue-on shoe to one hindlimb. Changes in Pmin and Pmax with induced hindlimb length difference were assessed with a two-way repeated-measures ANOVA with trial (straight, lunge with inside limb elevation, lunge with outside limb elevation) and surface (hard, soft) as within-subject factors. Change in Pmin, indicating an impact-type lameness, in the hind limb with the elevation, was significant in both the straight line and while lunging on both hard and soft surfaces. Change in Pmax, indicating pushoff-type lameness, in the opposite, non-elevated hind limb, was significant when trotting in a straight line but not while lunging.

## Introduction

Measurement of asymmetrical vertical movement of the pelvis is a common and accepted method to detect hindlimb lameness. The pelvis falls to a minimum height during, and rises to a maximum height after, stance, twice during one stride[1–5]. In horses without lameness this repetitive rise and fall is generally symmetrical in amplitude between right and left weight-bearing portions of the stride. In horses with pain during hindlimb weight-bearing, the pelvic fall, the pelvic rise, or both, decrease for the lame compared to the non- or less-lame hindlimb, reflecting decreased force x time on the limbs in the first (impact) or second (pushoff) parts of stance[1]. This results in higher minimum pelvic height during stance and/or lower maximum pelvic height after stance of the lame, or more lame, hindlimb. This model of hindlimb lameness detection and measurement assumes equivalent overall hindlimb length between right and left sides during normal weight bearing.

But, it is reasonable to assume that not all horses will have equal hindlimb length, even if the horse is healthy and functioning normally. Hindlimb length difference can result from growth discrepancies of the hooves or long bones between the right and left limbs. These discrepancies may have been caused by injuries that are healed and no longer painful, or differences in functional demand from specialized training or repetitive movement, leading to the development of a type of “leggedness” similar to “handedness” in humans[6, 7]. Such differences in hindlimb length would not be expected to cause pain, but the resulting differences in pelvic height may be measured as pelvic height asymmetry and assessed as hindlimb lameness. Is lameness measured in horses with different hindlimb length due to pain or simply to the difference in length? Can lameness measurement be affected by inducing differences in hindlimb length?

Differentiating lameness caused by hindlimb length difference or pain has important diagnostic and therapeutic ramifications. The effect of hindlimb length difference induced by hoof height elevation on equine hindlimb lameness has been initially studied by Vertz, et al. in 2018, where it was found that minimum and maximum pelvic height differences, as measures of hindlimb lameness, were significantly influenced in horses trotting in a straight line. However, in this study the effect of hindlimb length difference was only evaluated in horses trotting in a straight line on hard surfaces, limited conditions compared to common clinical evaluation [8].

The purpose of this study was to investigate the effect of induced hindlimb length difference on differences in minimum and maximum pelvic heights in horses trotting in a straight line and lunging on both hard and soft surfaces. We expected that, after induction of hindlimb length difference, differences in minimum and maximum pelvic height would reflect simple differences in limb length, with higher minimum and maximum heights in the limb with increased length. This would result in a false measurement of impact type hindlimb lameness (differences in minimum pelvic height) in the limb with increased length, and of a false pushoff type hindlimb lameness (differences in maximum pelvic height) in the limb opposite the limb with increased length, under all conditions (straight/lunge, hard/soft surfaces).

## Materials and methods

### Animals

Fifty horses used in an equestrian program and owned by a private university were evaluated for potential inclusion in this study. The William Woods Veterinary Ethics Committee governing the care of included horses approved use of horses in this study. All horses were being cared for and ridden daily by disciplined equestrian trainers and college students. Medical care was monitored and directed by a resident faculty equine veterinarian specialized in equine practice with over 20 years of experience evaluating lameness in horses (PS). Screening for enrollment in the study was initiated six months prior to the anticipated start date. Only horses that were not considered lame by simple subjective observation by the resident veterinarian, that did not measure with consistent hindlimb lameness, and that did not have existing tubera coxae height asymmetry while standing squarely were evaluated for enrollment. Throughout and after the study the horses continued to be used routinely in the equestrian program.

### Lameness measurement technique

Horses were instrumented with a body-mounted inertial sensor system (BMIS) consisting of a head, pelvic, and right forelimb sensors (Equinosis Q with Lameness Locator). The sensors on the head and pelvis measure vertical acceleration and convert to vertical position using an error-correcting double integration technique [9]. The right forelimb sensor, placed on the dorsum of the right pastern, measures angular velocity and is used as a time index marker indicating beginning and end of right forelimb stance. This time index is used to determine side of lameness as positive or negative differences in minimum and maximum pelvic height. In previous studies, the design of the sensors, methods of signal processing, wireless transmission, stride selection, and choice of hindlimb lameness measures have been described and validated [1,3–5, 9].

**Fig 1:**
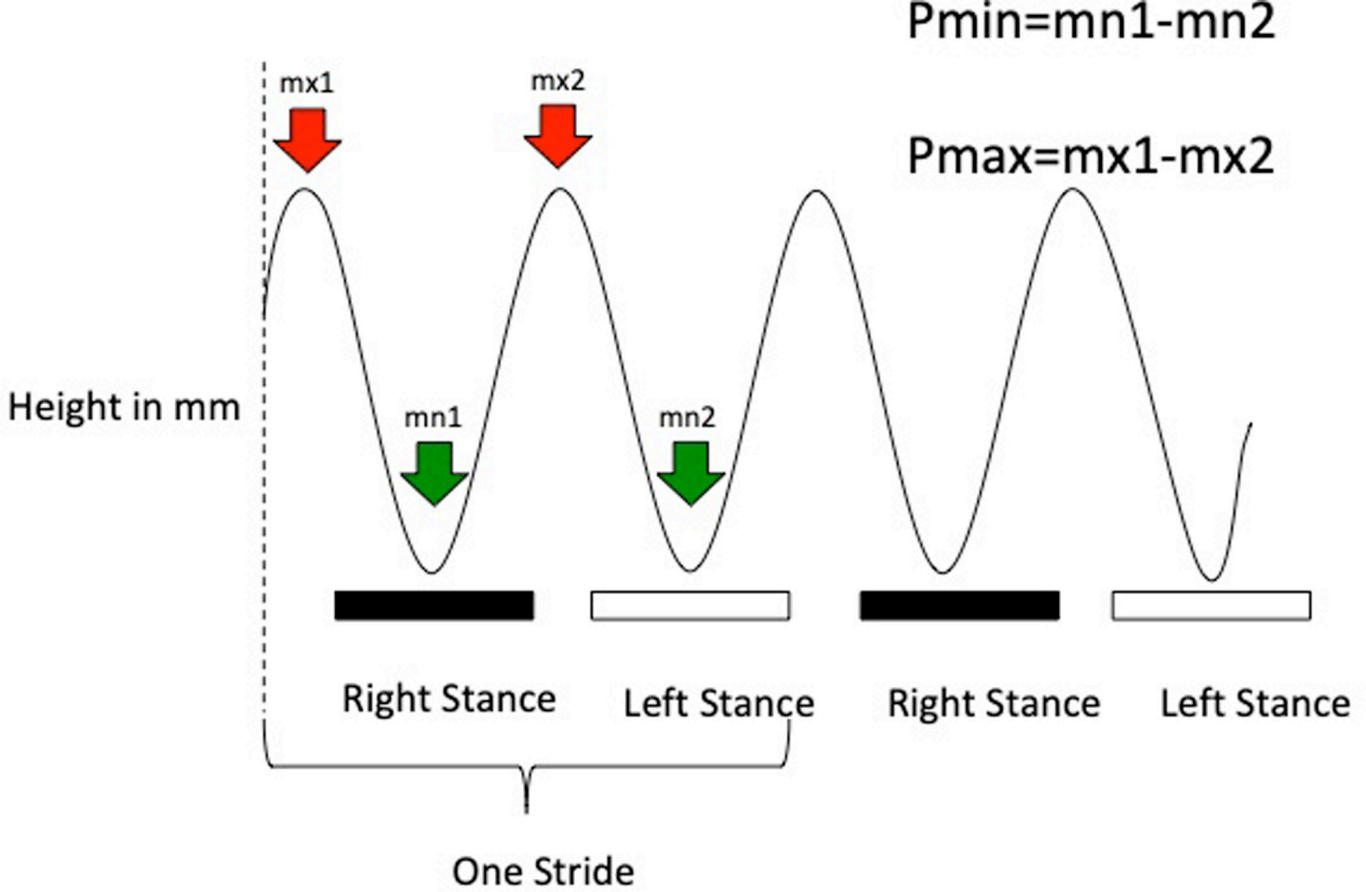
Schematic of pelvic height trajectory pattern of a normal horse. The solid black bars indicate timing of right hindlimb stance and the white bars timing of left hindlimb stance. P_min_ is determined by subtracting minimum pelvic height during left hindlimb stance (mn2) from minimum pelvic height during right hindlimb stance (mn1). P_max_ is determined by subtracting maximum pelvic height fter right hindlimb stance (mx2) from maximum pelvic height before right hindlimb stance (mx1). P_min_ and P_max_ units are in millimeters (mm).

In this study, only pelvic sensor measurements of hindlimb lameness were evaluated. For each stride, the minimum (Pmin) and maximum (Pmax) pelvic height differences between left and right stride halves were calculated (Fig 1). Pmin measures the result of decreased downward force onto a lame hindlimb in the first half of stance. Pmax measures the result of decreased upward force from a lame hindlimb in the second half of stance. By convention, positive Pmin and Pmax values indicate right hindlimb, and negative Pmin and Pmax indicate left hindlimb lameness. Pmin measures the lameness in the first half of stance (an impact type lameness). Pmax measures the lameness in the second half of stance (a pushoff type lameness).

A random number generator was used to determine if the control (before induction of hindlimb length difference) or treatment (after induction of hindlimb length difference) trials were collected first. The subsequent trials, treatment if the control was collected first, or control if treatment was collected first, were collected the same afternoon, or if not possible due to time constraints, the following day. Some trials were collected on separate days also to minimize the perceived effect of fatigue seen in some horses. Prior to collection, horses were lightly walked or lunged for 5–10 minutes. Hindlimb lameness was measured before and after induction of hindlimb length difference during a straight-line trot and lungeing in both directions on both hard (dirt) and soft (sand/fiber) surfaces. All horses had previously been trained to lunge and did so routinely as part of the equestrian program. Lungeing was performed in the same confined areas and by the same handler who had experience in both lungeing and working with the included horses. All horses were lunged in 8-m radius circles at a speed consistent with a working trot for the horses’ discipline. For straight line trot trials, the horse was trotted 90 meters back and forth, twice. This resulted in the collection of data for at least 25 trotting strides. Data was collected for lungeing trials continuously for about 1 minute. This resulted in the collection of data for at least 40 trotting strides. Speed of movement before and after hindlimb elevation was controlled by measurement of stride rate, keeping stride rate for both before and after elevation trials within a +/- 0.2 strides per second.

### Induction of hindlimb length difference

The Easyboot Glue-On shoe (EasyCare, Inc. Tuscon, AZ, US), with a ground surface thickness of 12.5 mm, was used to induce hindlimb length difference. The side of hindlimb length elevation was selected via a random number generator. The shoe was secured to the hind foot using Elastikon® 3-inch elastic tape. Nothing was applied to the other hindlimb.

To estimate the amount of pelvic asymmetry caused by induced hindlimb length differences, pelvic height from the ground was measured on both the left and right sides, before and after induction of hindlimb length difference. This was measured (as performed in a previous study) as the vertical distance from the ground to the tuber coxae using a tape measure with units of +/- 0.1 cm. [10]. During the measurement all horses were assessed to be standing square in the forelimbs and hindlimbs with weight-bearing on both hindlimbs on the same flat and level surface by the same investigator (JP).

### Animals

Sixteen adult (mean age = 15.8 years, range 8–25 years) horses (4 Warmbloods, 4 Thoroughbred/Thoroughbred crosses, 3 Quarter Horses, 3 Arabian/Arabian crosses, and 1 Morgan; 10 geldings, 6 mares), out of the 50 screened were included in the study. Six of the horses performed as hunter/jumpers, 6 were western performance horses, and 4 performed as dressage horses. All excluded horses measured with more than weak evidence of mild hindlimb lameness. None of the horses were excluded for pre-existing tuber coxae height asymmetry. The elevation was applied to the left hindlimb in 10 horses and the right hindlimb in 6 horses.

### Statistical analysis

To maximize power for the small subject numbers in a specific group, we pre-processed the data as follows. Elevation of the left and right hindlimb were combined for straight line trotting by multiplying the difference in Pmin and Pmax between before and after hindlimb length elevation by -1. This results in positive changes in the lameness measure to indicate lameness in the elevated limb, and negative changes in the lameness measure to indicate lameness in the non-elevated limb. Lunging trials were combined into inside limb and outside limb elevation groups by multiplying all left lunge trials by -1. This results in positive changes in the lameness measure to indicate lameness in the inside limb and negative changes in the lameness measure to indicate lameness in the outside hindlimb. Changes in Pmin and Pmax with induced hindlimb length difference were assessed using a two-way repeated-measures ANOVA with trial (straight, lunge with inside limb elevation, lunge with outside limb elevation) and surface (hard, soft) as within-subject factors. Diagnostic checks on the residuals were carried out to check normality and constant variance assumptions. Multiple comparisons were carried out by using the t-test statistic with Bonferroni adjusted p-values. Significance level was set at 0.05.

## Results

### Induction of hindlimb length difference and pelvic asymmetry

We could not obtain accurate measurements of the distance between the ground and both tubera coxae before and after induction of hind limb length differences. Horses varied greatly in tendency to lean toward or away from the side of elevation. Also, in horses with large gluteal musculatures, the top of the tubera coxae was obscured, affecting the accuracy of the measurement. However, in all horses, it was apparent, despite these difficulties, that the height from ground to tubera coxae increased (approximately 50 mm) in the elevated limb, and was unchanged in the non-elevated limb.

### Effect on lameness measures

Results of hind limb elevation are summarized in Table 1 for Pmin and in Table 2 for Pmax. There was significant change in Pmin and Pmax after elevation when the horse was trotting in a straight line on both hard and soft surfaces. Pmin increased to indicate (impact) lameness in the elevated limb. Pmax increased to indicate (pushoff) lameness in the non-elevated hindlimb. The amplitude of the mean change in Pmin (~ 6 mm) was greater than the amplitude of the mean change in Pmax (~ 3 mm).

**Table 1:** Mean (sd) and median change in Pmin values after elevation.

Direction	Surface	Mean (+/sd) change in Pmin after elevation (mm)	Median change in Pmin after elevation (mm)	P value
Straight	Hard	6.2 (4.6)	6.6	**0.017**
Straight	Soft	6.7 (3.4)	6.0	**< 0.0001**
Lunge (inside limb elevation)	Hard	5.4 (16.0)	3.0	**0.038**
Lunge (inside limb elevation)	Soft	4.9 (5.0)	4.1	**0.001**
Lunge (outside limb elevation)	Hard	-7.0 (4.8)	-5.4	**0.008**
Lunge (outside limb elevation)	Soft	-8.5 (6.8)	-6.4	**< 0.0001**

Bolded values indicate statistical significance. For straight line trotting, positive changes indicate an increase in the lameness measure in the elevated limb, and negative changes indicate an increase in the lameness measure in the non-elevated limb. For lunging, positive changes indicate an increase in the lameness of the inside limb, and negative changes indicate an increase in the lameness measure in the outside limb.

**Table 2:** Mean (sd) and median change in Pmax values after elevation.

Direction	Surface	Mean change in Pmax after elevation (mm)	Median change in Pmax after elevation (mm)	P value
Straight	Hard	-3.1 (6.9)	-3.4	**0.042**
Straight	Soft	-3.3 (4.4)	-4.4	**0.033**
Lunge (inside limb elevation)	Hard	-1.3 (9.3)	-2.7	0.389
Lunge (inside limb elevation)	Soft	-0.6 (3.5)	-1.1	0.691
Lunge (outside limb elevation)	Hard	1.7 (5.7)	1.7	0.268
Lunge (outside limb elevation)	Soft	1.2 (4.2)	1.7	0.419

Bolded values indicate statistical significance. For straight line trotting, positive changes indicate an increase in the lameness measure in the elevated limb, and negative changes indicate an increase in the lameness measure in the non-elevated limb. For lunging, positive changes indicate an increase in the lameness of the inside limb, and negative changes indicate an increase in the lameness measure in the outside limb.

During lunging Pmin changed significantly after elevation, but Pmax did not. Pmin increased indicating (impact) lameness in the elevated hindlimb on both hard and soft ground and when the elevation was on the inside and outside hindlimb.

## Discussion

This study investigated the effect of induced hindlimb length difference, and associated induction of pelvic asymmetry, on differences in minimum and maximum pelvic heights as measures of hindlimb lameness in horses trotting in a straight line and lungeing on both hard and soft surfaces. Difference in minimum pelvic height during stance, indicating an impact-type lameness, in the limb with the elevation, was consistently measured in both the straight line and while lungeing on both hard and soft surfaces. Difference in maximum pelvic height, indicating pushoff-type lameness in the opposite, non-elevated limb, was found only when the horse was trotting in a straight line.

Our results, using 12.5 mm in hindlimb elevation, are in agreement with another study, where only straight line trotting was evaluated, and hindlimb elevations were greater (15 and 30 mm). In both studies elevation of a hindlimb caused the local minimum of pelvic height during stance of that hindlimb to be higher than before the elevation. However, in both studies, the magnitude of the mean change in Pmin was less the actual height of the elevation applied to the hindlimb. We measured a mean change in Pmin of about 6 mm with a shoe elevation of 12.5 mm, while Vertz et al measured a change in mean Pmin ranging from 2 mm to 6 mm for an elevation of 15 mm, and 8–9 mm for an elevation of 30 mm. Similarly, we measured a mean change in Pmax of about 3 mm with a shoe elevation of 12.5 mm, while Vertz et al. measured a change in mean Pmax ranging from 1 mm to 4 mm for elevations of 15 and 30 mm. It should be noted that Vertz, et al. used non-standardized values of Pmin and Pmax and the system we used standardizes Pmax and Pmin to the individual horse’s total expected vertical pelvic movement, so that effects from very large or very small horses (or those with unusually large or small normal vertical pelvic movement) do not skew combined results. Pmax and Pmin are standardized to the individual horse by dividing by the expected vertical displacement of the pelvis in that stride (the second harmonic after removing nonperiodic movement), and then multiplied by a constant to keep the measurement in units of mm. The slight differences in results between our study and Vertz, et al. could be because of this.

Both studies show that the effect size for Pmax causing apparent pushoff lameness in the non-elevated limb is less than that for Pmin causing apparent impact lameness in the elevated limb. These results imply that the horse must be compensating by pushing off harder on the short limb compared to the limb that was elevated, so that the pelvis rises further upward after the foot of that limb leaves the ground. It also makes sense that Pmax would be less dependent than Pmin on absolute hindlimb length since Pmax is measured when neither hindlimb is in stance and Pmin is measured when one hindlimb is in stance.

Pmax was not changed significantly at the lunge whether the hindlimb length elevation was on the outside or inside hindlimb on either soft or hard ground. When the horse is lungeing on hard ground, the amount of pelvic rise after pushoff of the inside hindlimb is normally less than that of the outside hindlimb[11–14], which could mask or minimize the effect of inside hindlimb elevation (decreased pushoff on the non-elevated outside hindlimb). It is also possible that, as suggested for straight line trot, for both inside and outside limb elevations, the horse may be compensating by pushing off harder on the non-elevated limb, so the pelvis rises more than expected on the limb opposite the one with increased length.

### Practical relevance

Objective measurement of vertical pelvic movement asymmetry to assess hindlimb lameness may be subject to error if there is a pre-existing reason for asymmetric pelvic height, like differences in hindlimb length. Results of this study suggest that, if the nature of hindlimb lameness is primarily lack of pushoff, i.e. with significant amplitude of Pmax, the cause is less likely due to a pre-existing limb length asymmetry, but to actual differences in pushoff force between the hindlimbs. However, if the nature of the hindlimb lameness is primarily lack of impact, i.e. with significant amplitude of Pmin, at least part of the cause may be due to a pre-existing hindlimb length difference.

When using inertial sensors placed on midline to measure hindlimb lameness, if the measured hindlimb lameness is of a purely impact nature, one should check for pre-existing hindlimb length differences and pelvic asymmetry. This may be done by carefully standing the horse squarely on both hindlimbs and observing from the rear. Large amplitudes of pre-existing hindlimb length difference or vertical pelvic asymmetry should be taken into consideration when assessing Pmin. If the impact lameness is on the side of the higher hemi-pelvis, the true lameness may be less than measured. If the impact lameness is on the side of the lower hemi-pelvis, the true lameness due to pain may be higher than measured. Therefore, horses with pronounced pelvic asymmetry, may give the impression of a more severe lameness than actual, on the side of the higher hemi-pelvis, and of a less severe lameness than actual, on the side of the lower hemi-pelvis.

In human studies, the effects of limb length discrepancy on ground reaction forces are inconsistent, with some indicating increased impact force on the shorter limb[15, 16]. However, because humans are biped plantigrades and horses are quadruped unguligrades, and most human studies have been conducted at the walk, direct comparisons of results to this study are very difficult.

The long-term effect of compensation for limb length differences and pelvic asymmetry may be detrimental to the horse. Standardbred Trotters with hindquarter asymmetry had poorer racing performance than those without hindquarter asymmetry[10]. In human studies, leg length differences have been shown to increase the incidence of a variety of abnormalities and pathologies, including low back pain, hip osteoarthritis, sacroiliac disease, stress fractures, loosening of hip prostheses, and running injuries[17]. Whether these detrimental long-term effects are directly due to the asymmetry or to the compensatory efforts to adjust for this asymmetry are unknown.

### Limitations

The sample size of this study was relatively small, so only large changes in Pmin and Pmax were likely to be found. Due to concern by both owners and trainers involved in this study of the possibility of induced limb length difference adversely affecting overall health and performance in these horses that were in active training and use, the long term effect of hindlimb length difference could not be studied. It is possible that short-term compensation methods used by the horses would wane over a longer period of time. The small sample size and our restriction to studying only immediate effects prevents us from making generalizations of the effects of hindlimb length difference arising naturally over longer periods of time.

Horses with forelimb lameness will shift weight backward onto the contralateral hind limb during weight bearing, and they may pushoff less on the contralateral hind limb, affecting Pmax and Pmin. An existing forelimb lameness my cause a compensatory impact type ipsilateral hind limb lameness (causing a positive Pmin for a right forelimb lameness and a negative Pmin for a left forelimb lameness) and/or compensatory pushoff type contralateral hindlimb lameness (causing a negative Pmax for a right forelimb lameness and a negative Pmax for a right forelimb lameness). Although we did not control for or eliminate horses for possible use in the study by measuring for forelimb lameness, no horses with observable forelimb lameness were selected for use and no horses developed observable forelimb lameness during the study.

In this study we measured asymmetric vertical pelvic movement aligned within the horse-centered global axes system (vertical acceleration is acceleration normal to the surface of the device placed on the horse) with sensors placed on the midline of the horse’s body. We did not measure pelvic rotation that currently requires placing sensors off midline, on both sides of the body. It is possible that hindlimb length asymmetry does not create the same artefactual effect (a measured lameness caused only by limb length asymmetry) on measures of hindlimb lameness associated with pelvic rotation (hip hike, hip dip). Hip hike was not affected when elevation was placed on one hindlimb and horses were evaluated trotting in a straight line (8).

## Conclusions

This study has investigated the effect of induced hindlimb length difference on differences in minimum and maximum pelvic height measures of hindlimb lameness in horses trotting in a straight line and lungeing on both hard and soft surfaces. Pmin, the measure of impact-type hindlimb lameness, was consistently affected, increasing on the side of elevation in the straight and at the lunge. Pmax_,_, the measure of pushoff-type hindlimb lameness, was affected, but to a lesser degree than Pmin, during straight line evaluation, but not during the lunge.
